# Microwave Imaging under Oblique Illumination

**DOI:** 10.3390/s16071046

**Published:** 2016-07-06

**Authors:** Qingyang Meng, Kuiwen Xu, Fazhong Shen, Bin Zhang, Dexin Ye, Jiangtao Huangfu, Changzhi Li, Lixin Ran

**Affiliations:** 1 Laboratory of Applied Research on Electromagnetics (ARE), Zhejiang University, Hangzhou 310027, China; mengqingyang@zju.edu.cn (Q.M.); kuiwenxu@hdu.edu.cn (K.X.); sfz@zju.edu.cn (F.S.); aem@zju.edu.cn (B.Z.); desy@zju.edu.cn (D.Y.); huangfujt@zju.edu.cn (J.H.); 2 Department of Electrical and Computer Engineering, Texas Tech University, Lubbock, TX 79424, USA; changzhi.li@ttu.edu

**Keywords:** inverse scattering problem, microwave imaging, SOM, oblique incidence

## Abstract

Microwave imaging based on inverse scattering problem has been attracting many interests in the microwave society. Among some major technical challenges, the ill-posed, multi-dimensional inversion algorithm and the complicated measurement setup are critical ones that prevent it from practical applications. In this paper, we experimentally investigate the performance of the subspace-based optimization method (SOM) for two-dimensional objects when it was applied to a setup designed for oblique incidence. Analytical, simulation, and experimental results show that, for 2D objects, neglecting the cross-polarization scattering will not cause a notable loss of information. Our method can be potentially used in practical imaging applications for 2D-like objects, such as human limbs.

## 1. Introduction

The inverse scattering problem has been a popular topic in the microwave society for many years. Solving inverse scattering problems leads to diverse microwave imaging applications, such as non-destructive detection [1,2,3,4,5], medical examination [6,7,8,9,10,11,12,13,14], and through-wall imaging [15,16,17,18,19].

So far, various algorithms for inverse scattering problems have been studied [20,21,22,23,24,25,26,27,28,29,30,31,32,33,34,35]. Examples include the distorted Born iterative method [20], contrast source inversion method [21], the Gauss-Newton-type method [22], and stochastic-type inversion methods [23,24], and the recently proposed subspace-based optimization method (SOM) [27]. Among these algorithms, the SOM has been attracting many interests due to its unique advantages of fast convergence and insensitivity to noise [27,28,29,30,31,32,33,34,35]. The SOM performs spectrum analysis on the scattering operator, mapping from the induced source to the scattered field. Using the spectral property of the scattering operator, the SOM first determines a part of the induced source and then obtains the rest through optimization. Such a procedure simplifies the nonlinear optimization problem, stabilizes the solution, and accelerates the convergence speed. The SOM has been proposed for solving two-dimensional (2D) inverse scattering problems in both the transverse magnetic (TM) [27] and the transverse electric (TE) [28] scenarios. Moreover, it was extended to solve inverse scattering problems for perfectly electric conducting scatterers [29,30] and three-dimensional (3D) inverse scattering problems [31]. Meanwhile, extended SOM-based methods, such as TSOM [32], FFT-TSOM [33], and MR-FFT-TSOM [34], have been proposed to reduce the computational complexity, regularize the nonlinear inverse problem and improve the quality of imaging. Both numerical simulation and experimental results have validated the effectiveness of the SOM [33,35]. In recent studies, there are also results concerning “virtual experiments frameworks” [36,37]. Using the linear relationship between the incident and the scattered field, virtual experiments can be designed and special convenient conditions can be enforced. Thus, advantages can be gained from such knowledge. The inverse scattering technique is also widely used in ground penetrating radars (GPRs). GPRs are non-destructive imaging systems that can provide images of the subsurface. An overview of the microwave imaging strategies can be found in [38].

**Figure 1:**
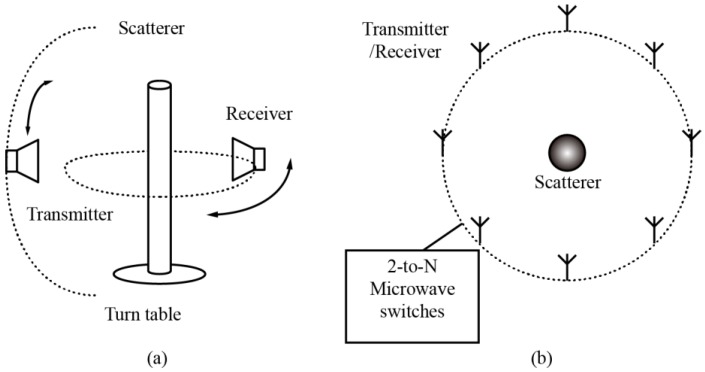
Typical setups for inverse scattering problem based microwave imaging. (**a**) Mechanical turning system; and (**b**) an electronically-controlled system with a 2-to-N microwave switching network.

Apart from inversion algorithms, different experimental setups for measuring scattered fields have also been proposed [39,40,41,42,43], based on which experiments were conducted to test the performance of such algorithms. These systems can be categorized into two typical types. Systems in [39,40] consist of a transmitting antenna, a receiving antenna, and some mechanical turning device, as described in Figure 1a. Usually, the transmitter is fixed at an azimuth angle and the receiver is rotated in the horizontal plane to measure the scattered fields in different directions. The scatterers can also be turned by a turntable, which is equivalent to turning the transmitter in the horizontal plane. Using such a setup, researchers are able to obtain multiview scattered data of targets under test. When this setup is used to measure 2D targets under normal far-field incidence, the receiving antenna could interfere, or even block the incident field, producing errors in the incidence modeling. Being a mechanical turning system, it also suffers from a long measurement period.

Systems in [41,42,43] consist of a fixed antenna array, whose elements are uniformly placed on the perimeter of a circle and a complicated bi-directional microwave switch matrix, as described in Figure 1b. The switch matrix connects the elements to the measurement ports of microwave instruments, such that each antenna element can be switched to work in either transmitting or receiving mode. The advantage of such a setup is that the whole system does not have any mechanical turning device. Instead, it eliminates the complexity of physically turning the scatterers and antennas by electronic control and system calibration. So far, this kind of system is mainly used in near-field measurements.

**Figure 2:**
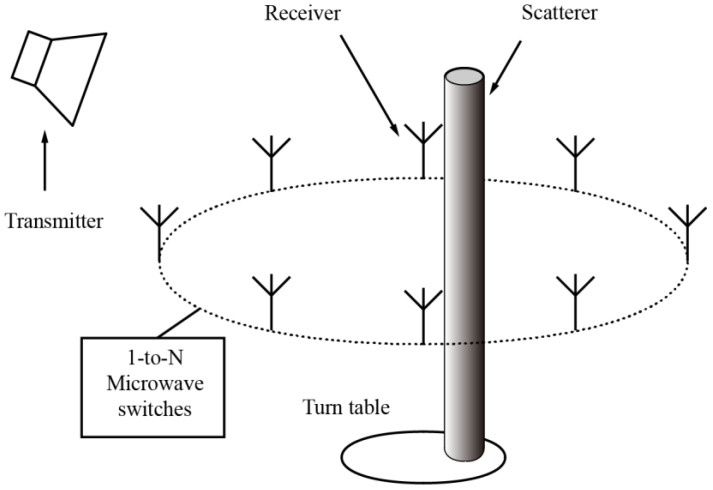
Proposed experimental setup with an oblique incidence and a 1-to-N microwave switching network.

In this paper, we experimentally investigate the performance of conventional SOM for 2D objects when it was applied to a setup designed for oblique incidence, as shown in Figure 2. This setup is able to combine the merits of the existing setups by using only one transmitting antenna and reducing the switching network to a 1-to-N one. In this case, the system calibration can be simplified, the transmitting antenna can be conveniently placed to ensure a plane wave incidence, and the detecting range can be independently optimized. Obviously, improved system calibration and reduced errors in incidence modeling and detected data will help to obtain better imaging results. However, the precondition to employ this setup is that the oblique incidence should not cause a notable loss of imaging quality. 

In this paper, we investigate the application of SOM for this specific setup, focusing on the impact of the imaging quality due to the oblique incidence. In order to avoid the use of dual-polarized receiving antennas, which will introduce technical difficulty in system integration and bring additional calibration, the TM-polarized oblique incidence is used, and only the TM-polarized scattering is detected. Analytical, simulation and experimental results show that for 2D objects, neglecting the TE-polarized (cross-polarization) scattering will not cause a notable loss of information and, therefore, the quality of microwave imaging is not degraded compared with the case under normal illumination.

This paper is organized as follows. Section 2 presents the modeling of the forward scattering problem and the inversion algorithm for 2D objects under oblique microwave illumination. To validate the proposed algorithm, numerical simulation and experimental measurements are conducted in Section 3 and Section 4, respectively. Finally, a conclusion is drawn in Section 5.

## 2. Algorithms

**Figure 3:**
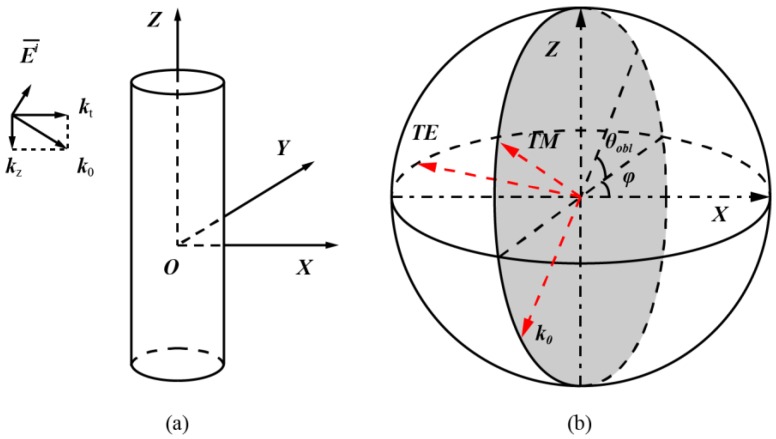
Problem description. (**a**) Analysis model of a two-dimensional object under oblique incidence; and (**b**) coordinate definition. It should be noted that the *TM* and *TE* components of the electric field and the wave vector *k*_0_ are orthogonal to each other.

In this Section, we focus on the modeling of the forward and inverse problem for the scattering of a 2D dielectric object under oblique incidence, as described in Figure 3a. So far, most of the studies on the inverse scattering problems are focused on 2D or 3D scenarios under normal incidence [20,21,22,23,24,25,26,27,28,29,30,31,32,33,34,35,36,37,38,39]. Research on imaging 2D objects under oblique incidence are rarely studied. Inverse scattering problems under oblique incidence can be considered as reduced 3D problems. However, in engineering practice where the scatterers can be approximated as 2D objects [44], modeling the scatterers with 3D meshes is much more complicated and makes the computation more time and memory consuming. In this case, 2D approximation can potentially lead to some advantages in practical applications. 

### 2.1. Forward Problem

There are many methods to obtain the solutions of forward problems [45,46,47,48,49]. Here we extend the method proposed in [45], using the inverse matrix, instead of the conjugate gradient method, to solve the equation we derived from the model of the forward scattering problem.

Consider the general equation that describes the scattering:

(1) $$\bar{E}\left(\bar{r}\right)={\bar{E}}^{i}\left(\bar{r}\right)-j\omega \bar{A}-\nabla \Phi$$

where $\bar{E}$ and ${\bar{E}}^{i}$ denote the total and incident electric fields. According to Equation (7) in [45], for an electric oblique incidence with wave number *k_z_* in the axial direction, as shown in Figure 2, the solution of the electric field for a 2D object is:

(2) $$\bar{E}\left(x,y\right)={\bar{E}}^{i}\left(x,y\right)+k_{0}^{2}\iint \bar{\bar{G}}\left(k_{t}\rho \right)\cdot \left\{\bar{\bar{\chi }}\left(x^{\prime },y^{\prime }\right)\cdot \bar{E}\left(x^{\prime },y^{\prime }\right)\right\}dx^{\prime }dy^{\prime }$$

where $\bar{\bar{G}}\left(k_{t}\rho \right)$ is known as the dyadic Green’s function.

For a scatter made of an isotropic medium, when the incidence is normal and axially polarized, Equation (2) turns into a single, uncoupled integral equation since the total electric field $\bar{E}$ only has the axial component. However, in the oblique incidence case, $\bar{E}$ consists of three components and Equation (2) should be considered as three coupled integral equations.

To numerically solve the integral equations, a discretization procedure is needed. Based on [45], a set of algebraic equations can be obtained as:

(3) $$\bar{E}={\bar{E}}^{i}+{\bar{\bar{G}}}_{D}\cdot \bar{\bar{\chi }}\cdot \bar{E}$$

The domain of interest is discretized into *N* = *M* × *M* square cells. In Equation (3), $\bar{E}$ and ${\bar{E}}^{i}$ are both 3*N* × 1 vectors, denoting the total and incident electric fields, respectively. ${\bar{\bar{G}}}_{D}$ is a 3*N* × 3*N* matrix. The elements of the discrete Green’s function, ${\bar{\bar{G}}}_{D}$, can be estimated according to [45]. It should be noted that in [45], a numerical integration procedure is recommended for the estimation of ${\bar{\bar{G}}}_{D}$. Nevertheless, the computational cost of our problem does not increase much since ${\bar{\bar{G}}}_{D}$ does not change when solving the inverse problem. $\bar{\bar{\chi }}$ is a 3*N* × 3*N* diagonal matrix. The first *N* diagonal elements of $\bar{\bar{\chi }}$ are given by $\chi_{m}=\varepsilon_{m}-1$, where $\varepsilon_{m}$ denotes the relative permittivity in cell *m*. The other diagonal elements of $\bar{\bar{\chi }}$ are given by $\chi_{m+2N}=\chi_{m+N}=\chi_{m}$.

Once ${\bar{E}}^{i}$, ${\bar{\bar{G}}}_{D}$, and $\bar{\bar{\chi }}$ are successfully constructed, $\bar{E}$ could be solved through a variety of methods. After the total electric field $\bar{E}$ is obtained, the scattered field is given by:

(4) $${\bar{E}}^{sca}={\bar{\bar{G}}}_{s}\cdot \bar{\bar{\chi }}\cdot \bar{E}$$

where ${\bar{E}}^{sca}$ is a 3*N_s_* × 1 vector that denotes the scattered field at $N_{s}$ observation points. ${\bar{\bar{G}}}_{s}$ is a 3*N_s_* × 3*N* matrix. The estimation of the elements of ${\bar{\bar{G}}}_{s}$ is similar to that of ${\bar{\bar{G}}}_{D}$, but the Green’s function is evaluated between *N* cells and the observation points.

Until now, the electric fields are described in a Cartesian coordinate system. It is more straightforward to represent the scattered electric field by the TE and TM components. Details of the polarization definition are described in Figure 3b. It should be noted that the TM and TE components of the electric field, as well as the wave vector *k_0_*, are orthogonal to each other. The TE-TM frame is used because TE and TM components are orthogonal. This makes it clearer in the simulation part when comparing among the results inversed with different components of scattered fields. A similar definition is also used in [46]. The transformation of coordinates is thus:

(5) $$\left[\begin{array}{l} E_{TE}^{sca}\\ E_{TM}^{sca} \end{array}\right]=\left[\begin{array}{ccc} \sin\left(\phi \right) & -\cos\left(\phi \right) & 0\\ 0 & 0 & 1/\cos\left(\theta_{obl}\right) \end{array}\right]\cdot \left[\begin{array}{l} E_{x}^{sca}\\ E_{y}^{sca}\\ E_{z}^{sca} \end{array}\right]$$

Here $E_{TE}^{sca}$ and $E_{TM}^{sca}$ denote the TE and TM components of the scattered electric fields, respectively. To combine this transformation with the derived algorithm, both sides of Equation (4) are left multiplied by the transformation matrix, which is equivalent to making a transformation to ${\bar{\bar{G}}}_{s}$. In the discussion below, we consider the scattered fields described by the TM and TE components without changing the form of the equations.

### 2.2. Inverse Problem

In this section, we discuss the inversion algorithm suitable for the imaging of 2D objects under oblique incidence based on the SOM.

The main idea of SOM is to determine a part of induced current by analyzing the spectral properties of the mapping from the induced current to scattered fields, and then obtain the rest part through optimization. Usually, the optimization procedure is to construct a cost function, and use a proper method to minimize it [27].

In our work, we construct the cost function following the method proposed in [27], i.e., using the singular value decomposition technique and choosing a truncation value *L*. It can be given by:

(6) $$f\left(\bar{\alpha },\bar{\bar{\chi }}\right)=\sum_{p=1}^{N_{i}}({\left\|{\bar{\bar{G}}}_{s}\cdot \left({\bar{I}}_{p}^{s}+{\bar{\bar{V}}}^{n}\cdot {\bar{\alpha }}_{p}\right)-{\bar{E}}_{p}^{sca}\right\|}^{2}/{\left\|{\bar{E}}_{p}^{sca}\right\|}^{2}+{\left\|\bar{\bar{A}}\cdot {\bar{\alpha }}_{p}-{\bar{B}}_{p}\right\|}^{2}/{\left\|{\bar{I}}_{p}^{s}\right\|}^{2})$$

where *N_i_* is the number of incidences, ${\bar{\bar{V}}}^{n}$ is composed of the last 3*N* − *L* right singular vectors, and ${\bar{\alpha }}_{p}$ is a 3*N* − *L* dimensional vector. ${\bar{I}}_{p}^{s}$ and ${\bar{E}}_{p}^{sca}$ denote the deterministic current and scattered field due to the *p-*th incidence, $\bar{\bar{A}}=\left({\bar{\bar{V}}}^{n}-\bar{\bar{\chi }}\cdot {\bar{\bar{G}}}_{D}\cdot {\bar{\bar{V}}}^{n}\right)$ and ${\bar{\bar{B}}}_{p}=\bar{\bar{\chi }}\cdot ({\bar{E}}_{p}^{i}+{\bar{\bar{G}}}_{D}\cdot {\bar{I}}_{p}^{s})-{\bar{I}}_{p}^{s}$. Various methods can be used to minimize this cost function. In our work, the method proposed in [27] is used, i.e., updating the coefficients ${\bar{\alpha }}_{p}$ through the conjugate gradient method and then updating the contrast $\bar{\bar{\chi }}$ by solving a quadratic minimization problem. 

In the 2D inverse scattering problem under normal TM incidence, ${\bar{\bar{G}}}_{s}$ is a $N_{s}\times N$ matrix; while under oblique incidence, ${\bar{\bar{G}}}_{s}$ is a $2N_{s}\times 3N$ matrix if the scattered field is decomposed into TM and TE components. ${\bar{\bar{G}}}_{s}$ can be written as ${\bar{\bar{G}}}_{s}=\left[{\bar{\bar{G}}}_{s,TM};\;{\bar{\bar{G}}}_{s,TE}\right]$, where ${\bar{\bar{G}}}_{s,TM}$ is a $N_{s}\times 3N$ matrix which denotes the mapping from the induced current to the TM component of the scattered field, and ${\bar{\bar{G}}}_{s,TE}$ is a $N_{s}\times 3N$ matrix which denotes the mapping from the induced current to the TE component of the scattered field. In the following discussion about the influence of neglecting some components of the scattered fields, when full polarization data is used, ${\bar{\bar{G}}}_{s}$ is considered; when only TM polarization data is used, only ${\bar{\bar{G}}}_{s,TM}$ is considered; when only TE polarization data is used, only ${\bar{\bar{G}}}_{s,TE}$ is considered.

It should be noted that an oblique incidence may affect the imaging quality due to the fact that the main variable in the Green function is $k_{t}\rho$, which is different from $k_{0}\cdot \rho$ in the normal incidence scenario [45]. Unlike $k_{0}$, $k_{t}$ changes with the angle of oblique incidence. The change of the Green function and the incident field may affect the imaging performance.

## 3. Numerical Simulations

In this Section, we present numerical simulations to test the performance of the SOM when it is applied to the proposed setup with oblique incidence. 

**Figure 4:**
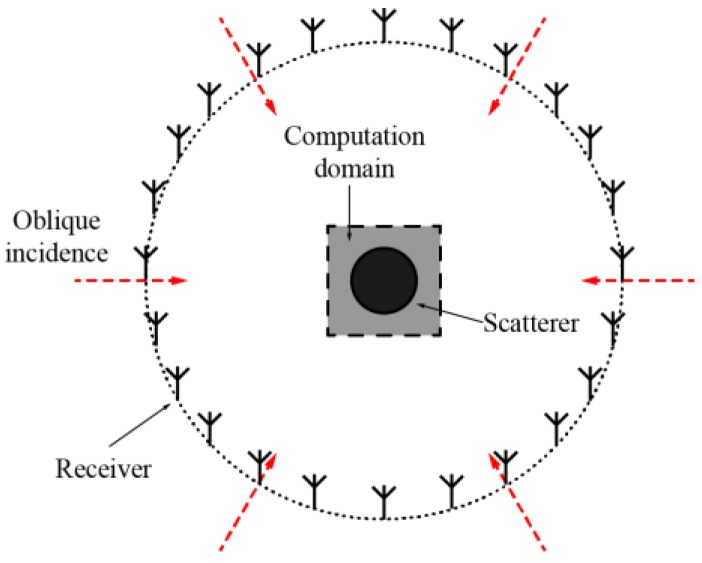
Simulation setup on the x-y plane.

The setup for the simulation is shown in Figure 4. Six TM-polarized plane waves whose propagation directions are oblique and equally spaced on a circle are used as incidences, and 24 linearly-polarized antennas placed on a horizontal circle are used to detect the scattered field. The computation domain is set as a square area of 25×25 cm^2^, which is discretized into a 40×40 mesh grid. In the simulations, the frequency of the incident wave is 2.4 GHz, whose corresponding wavelength λ in free space is 12.5 cm. The background is set as air. 

The scatterer is a dielectric cylinder whose relative permittivity is 3. The radius of the cylinder is 0.2λ, i.e., 2.5 cm. The cylinder is firstly positioned at the coordinate (0, 0) and later at (5cos30°, −5sin30°). The units are both cm. In the inversion procedure, the simulated scattered field is introduced with an additive white Gaussian noise, such that the signal to noise ratio (SNR) of the scattered field is 10 dB. The singular value truncation number *L* is chosen as 25. It is chosen so that singular values can notably change the slope in the spectrum. Note that the spectrum varies with the angle of oblique incidence, so the smallest “knee” is chosen from scenarios with different incident angles. In our numerical simulations, the only prior information we used is that the scatterer in the computational domain is dielectric and, thus, has a positive contrast, i.e., the relative permittivity of the scatterer is greater than or equal to 1.

**Figure 5:**
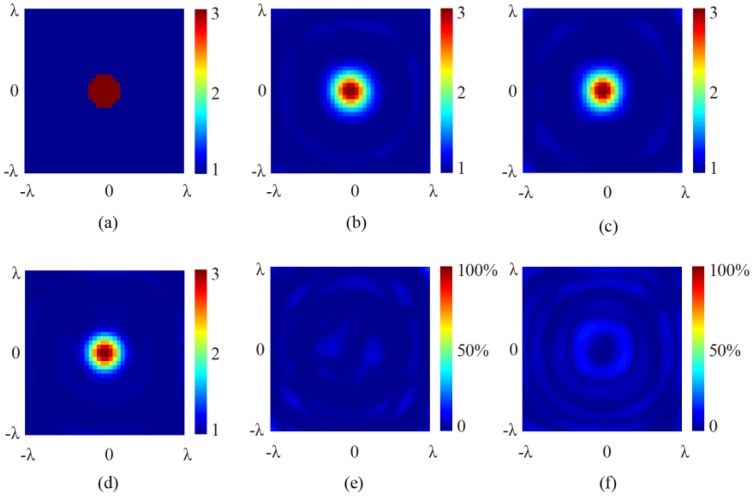
The retrieved cross-section profile of the centered 2D cylinder. (**a**) Original profile; (**b**) retrieved result at normal incidence; (**c**) retrieved result at 15° angle of incidence; (**d**) retrieved result at 30° angle of incidence; (**e**) relative error between 5c and 5b; and (**f**) relative error between 5d and 5b.

**Figure 6:**
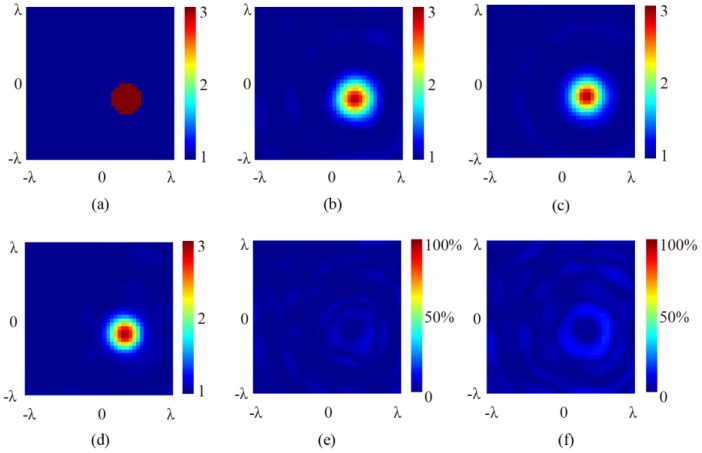
The retrieved cross-section profile of the off-centered 2D cylinder. (**a**) Original profile; (**b**) retrieved result at normal incidence; (**c**) retrieved result at 15° angle of incidence; (**d**) retrieved result at 30° angle of incidence; (**e**) relative error between 6**c** and 6b; and (**f**) relative error between 6d and 6b.

**Figure 7:**
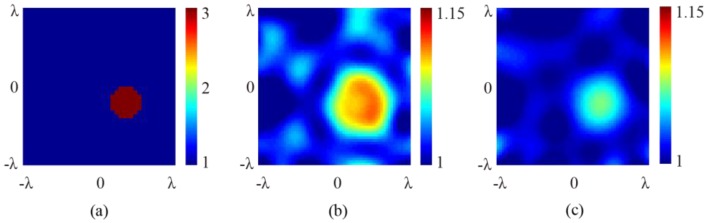
The retrieved cross-section profile of the off-centered 2D cylinder using first order Born approximation. (**a**) Original profile; (**b**) retrieved result at 15° angle of incidence; and (**c**) retrieved result at 30° angle of incidence.

Figure 5 and Figure 6 show the results of the numerical simulations obtained by the SOM algorithm. The original cross-section profiles of the target are illustrated in Figure 5a and Figure 6a, in which the 2D cylinder is placed at the center and off-center locations, respectively. Note that the profile has been meshed and the outline is, therefore, not a circle. It is seen that for all of the different positions of the target and the incidence angles of 0° (normal incidence), 15°, and 30°, the retrieved results of the relative permittivity, the location, and the cross-section profile of the target comply well with each other. To compare the difference between the inversed results under normal and oblique incidences, the relative error for each point in the image is calculated by:

(7) $$error_{1}=\left|\varepsilon_{oblique}-\varepsilon_{normal}\right|/\left|\varepsilon_{normal}\right|\times 100\%$$

and the error figures are given in Figure 5e,f and Figure 6e,f, respectively. It is seen that although ripples exist, the error remains very small in both the background and the scatterer regions, showing that the oblique incidence does not notably reduce information. It is worth noting that, for differently located targets, no notable differences exist between the results retrieved from the scattered fields under normal and oblique incidences. As a comparison, retrieved results using the first order Born approximation are shown in Figure 7. The truncated singular value decomposition (TSVD) method is used to solve the linearized inverse problem. It can be seen that in the off-center cylinder scenario, the scatterer can be identified. However, the permittivity is much lower due to the approximation. This indicates that the application of SOM is reasonable.

The simulation results shown in Figure 5 and Figure 6 clearly validate the proposed algorithm. However, the above simulations used both the TE and the TM polarization information of the scattered field. In many practical cases, measuring the full polarization data is difficult, it may make the measurement setup complex and result in being much more time consuming in the inversion. To simplify the measurement in practice, we further test the proposed algorithm with part of the polarization of the scattered field. Since the incident wave is TM polarized in our simulation setup, and in the experimental setup later, we will use the TM component of the scattered field to retrieve the spatial distribution of the scatterer’s relative permittivity.

**Figure 8:**
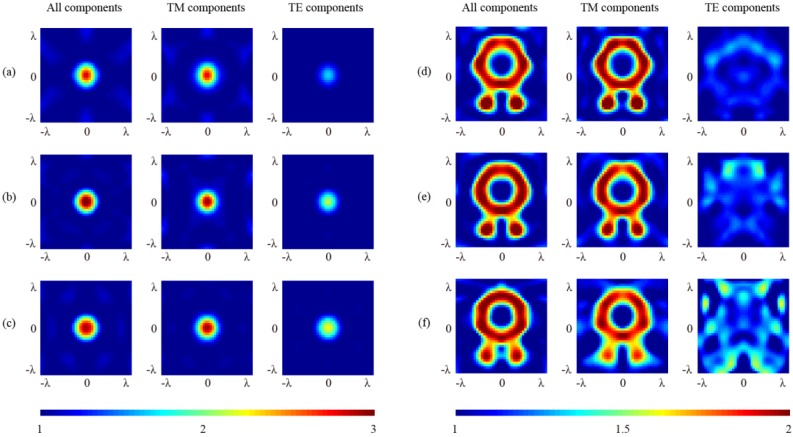
The retrieved cross section profiles of the 2D cylinder using different polarization components of the scattered field. (**a**) 15° angle of incidence; (**b**) 30° angle of incidence; and (**c**) 45° angle of incidence. The retrieved cross-section profiles of the 2D “Austria” profile using different polarization components of the scattered field; (**d**) 15° angle of incidence; (**e**) 30° angle of incidence; and (**f**) 45° angle of incidence.

Unlike the normal incidence scenario, the scattered field under oblique TM incidence also contains a cross-polarized TE component [46]. Therefore, the inversion with the TM component of the scattered field only means that the algorithm does not make full use of the scattered field data. In order to quantify the difference between inversion results with and without the full use of the polarization information, we further conduct more numerical simulations. The first simulation is the same as previous simulation based on the centered dielectric 2D cylinder. In the second simulation, a 2D scatterer with the so-called “Austria” cross-section profile is used [27], whose relative permittivity is set as 2. For both simulations, the inversion results solely rely on the TM and the TE polarization data, as well as the results using the full polarization information, are shown in Figure 8 for three different angles of incidence, i.e., 15°, 30°, and 45°. Since there are different unknowns in the TM and TE cases, in the optimization we used the same mesh and termination criterion for both cases, to make sure the comparison between them is reasonable. It is seen that in all cases, the profiles retrieved with the TM components are very similar to those retrieved with full polarization components. In the meantime, the retrieved profiles with the TE components have significant differences with the ones with both components.

To describe the difference between these cases, we define an error function as:

(8) $$error_{2}={\sum \left|\varepsilon_{inv}-\varepsilon_{all}\right|}^{2}/\sum {\left|\varepsilon_{all}\right|}^{2}\times 100\%$$

where $\varepsilon_{inv}$ and $\varepsilon_{all}$ denote the inversed profile with only the TM or the TE polarization data and with all polarization information, respectively, and the summation is performed in the computational domain. The computed errors are listed in Table 1. It is seen that within 45° angle of incidence, the inversion results using only the TM component are satisfactory, keeping the error less than 1% in all incidences. On the other hand, the inversion results using only the TE component are with much larger error, especially for the target with the “Austria” profile. The large difference between these two cases implies that neglecting TE component does not lead to a notable loss of imaging quality.

These simulation results also comply with existing theory [45]. The scattering Green function matrix ${\bar{\bar{G}}}_{s}$ in Equation (4) can be written in the form ${\bar{\bar{G}}}_{s}=\left[g_{xx},\;g_{xy},\;g_{xz};\;g_{yx},\;g_{yy},\;g_{yz};\;g_{zx},\;g_{zy},\;g_{zz}\right]$, where $g_{xy}$ denotes the mapping to the *x* component of the scattered field from the *y* component of the induced source, etc., according to the Equations (10)–(12) in [45], $g_{xz}=C\cdot \frac{\left(x-x^{\prime }\right)}{\rho }$ and $g_{yz}=C\cdot \frac{\left(y-y^{\prime }\right)}{\rho }$, where C is a constant. Since the receivers are in the far field, $\frac{\left(x-x^{\prime }\right)}{\rho }\approx \text{cos}\phi$ and $\frac{\left(y-y^{\prime }\right)}{\rho }\approx \text{sin}\phi$. Consider the coordinate transformation described by (12) in our paper, the elements corresponding to the mapping from *z* component of induced source to TE component of the scattered field can be written as $g_{xz}\cdot \text{sin}\phi -g_{yz}\cdot \text{cos}\phi \approx C\cdot \left(\text{sin}\phi \cdot \text{cos}\phi -\text{sin}\phi \cdot \cos\phi \right)=0.$ This indicates that the influence on the TE component of the scattered field from the *z* component of the induced source is weak under the precondition of that the incident field is TM polarized, Therefore, ignoring the TE component of the scattered field will not cause a notable degradation in the quality of the retrieved image.

The above simulation results and analysis strongly imply that in the case we discussed, i.e., scattering of 2D objects under oblique incidence, the TM component of the scattered field contains sufficient information for the retrieval of the scatterer, at least when the angle of incidence is within a limited range. In this case, imaging using the TM component only would not notably lose its quality. This conclusion can be used to simplify the measurement setup, as discussed in the following.

For even larger incidence angles, there is not much practical meaning since it is difficult for the measurement to be conducted in accordance with the approximation [50].

## 4. Experiments

In this Section, we perform experiments to verify the proposed algorithm.

### 4.1. Experimental Setups

**Figure 9:**
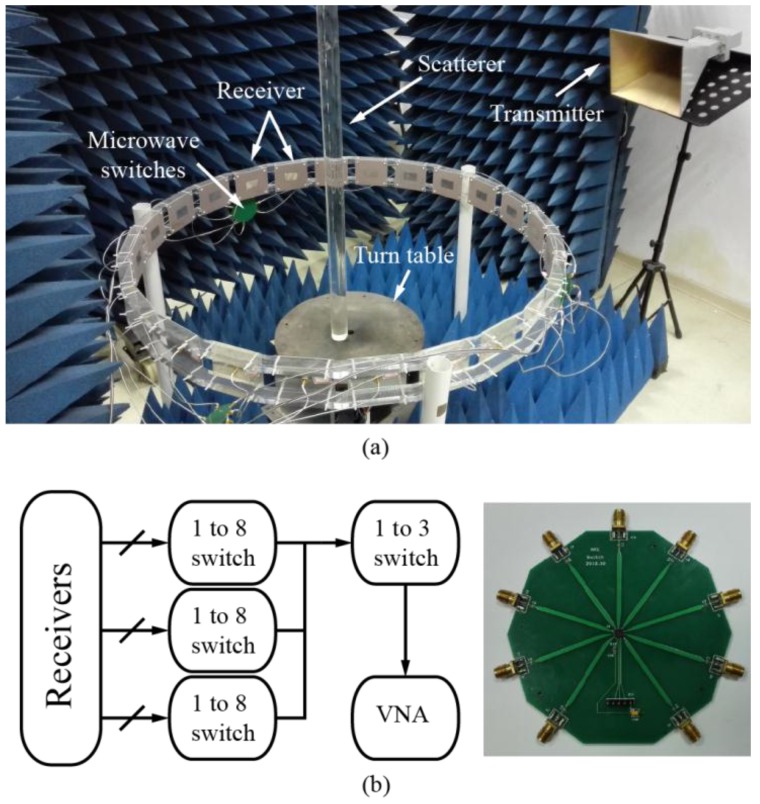
Experimental setup. Inset shows one of the three simple-structured unidirectional microwave switches. (**a**) Experimental setup; and (**b**) simple structured microwave switch.

Figure 9a shows the implemented experimental setup, which is very similar to the simulation model depicted in Figure 4. The system is designed to work at 2.4 GHz, whose corresponding wavelength λ in free space is 12.5 cm. The main part of the detecting device is an array of 24 linearly-polarized patch antennas. These antennas are evenly spaced on a circle with a diameter of 113 cm, meaning that the distance between the center of the computation domain and the antenna is around 4.5λ. All antennas can be switched through commercial integrated microwave switches (Hittite HMC321LP4, Hittite Microwave, Chelmsford, MA, USA) mounted on three printed circuit boards, as shown in Figure 9b, and can be controlled by a micro-controller unit (Atmel’s ATmega88, Atmel, San Jose, CA, USA). These patch antennas are used as receivers, and a standard horn antenna (Yinglian Microwave’s LB34015CSF, Yinglian Microwave, Chengdu, China) is used as the transmitter to provide the oblique illumination.

A vector network analyzer, Agilent’s 8722ES (Agilent, Santa Clara, CA, USA), is used to conduct the measurements. A wideband amplifier, Agilent’s 8449B, is used before the horn antenna to enhance the power level of the transmitted signal. Through switching between the receiving antennas, we can measure the scattered field at different angles. A turntable is placed in the center of the antenna array. By turning the object, we can also measure the scattered field at different incidences by using only one fixed transmitting antenna. In our experiment, the number of incidences is 6.

In the measurement, the electric field at each receiver’s position in the absence and in the presence of the target should be measured separately. Then, the turn table rotates the target by 60°, and the electric fields in the presence of the target can be measured. Such rotating and measurement should be repeated six times. Through calculating the difference between the measured fields in the presence and in the absence of the target, we can obtain a 6 × 24 matrix of measured data of scattered fields. Since 2.4 GHz is located in an open ISM band, before experiments it is necessary to make sure that no nearby wireless device is using this frequency.

It is seen that the above setup combines the advantages of the conventional setups shown in Figure 1a,b. While all antennas are fixed, the bi-directional microwave switch matrix can be replaced with simple-structured, unidirectional switches, as shown in the inset of Figure 9. These will bring significant convenience to the implementation and calibration of the measurement system.

### 4.2. Calibration of the Measurement System

**Figure 10:**
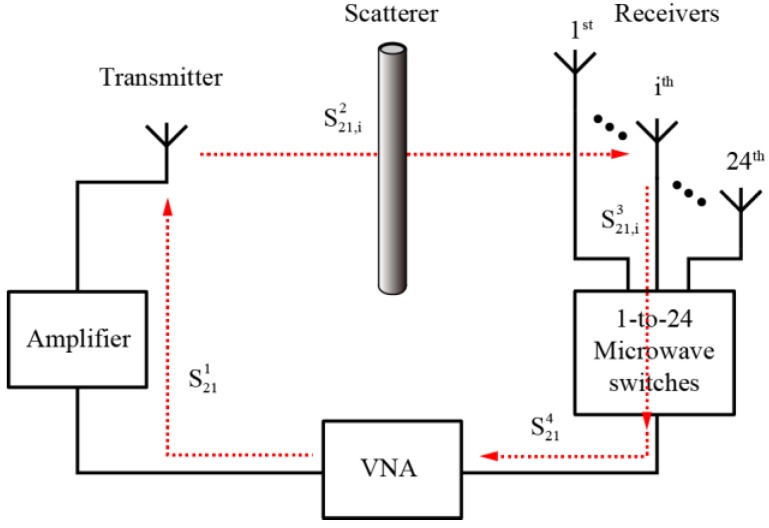
Calibration of the experimental setup.

Similar to all the other experimental setups, a precision calibration to the measurement setup is crucial to obtain satisfactory inversion results. In our work, we used a calibration method similar to that reported in [51]. The scattered field is calculated by subtracting the incident field from the total field. Measuring with the Vector Network Analyzer (VNA), we can obtain the $S_{21}$ parameter of the measurement system. The $S_{21}$ parameter of the entire system can be divided into four parts, i.e., $S_{21,i}=S_{21}^{1}\cdot S_{21,i}^{2}\cdot S_{21,i}^{3}\cdot S_{21}^{4}$, *i* = 1, …, 24. As shown in Figure 10, $S_{21}^{1}$, $S_{21,i}^{2}$, $S_{21,i}^{3}$, and $S_{21}^{4}$ denote the $S_{21}$ parameters from the VNA’s output port to the transmitting antenna, the transmitting antenna to the *i-*th receiving antenna, the *i-*th receiving antenna to the switch, and the switch to the VNA input port, respectively. For different paths, $S_{21}^{1}$ and $S_{21}^{4}$ are the same, $S_{21,i}^{3}$ can be measured, $S_{21,i}^{2}$ has a definite relationship with the electric field. Therefore, we can measure the relative value of the electric field by measuring the $S_{21}$ parameters of the measurement system.

In the calibration, the $S_{21}$ parameter for each transmitter-receiver pair in the absence and in the presence of the scatterer, denoted as ${\bar{E}}^{i}$ and ${\bar{E}}^{t}$ respectively, should be measured first. The obtained ${\bar{E}}^{i}$ and ${\bar{E}}^{t}$ are both a 24 × 6 matrix. Each column of ${\bar{E}}^{t}$, written as ${\bar{E}}_{p}^{t}$, *p* = 1, 2, …, 6, denotes the scattered field detected by 24 receivers due to the *p*^th^ incidence. Finally, the scattered field at different incidences can be given by ${\bar{E}}_{p}^{sca}={\bar{E}}_{p}^{t}-{\bar{E}}^{i}$.

It should be noted that the data we measured until now cannot be directly used to retrieve the scatterer. Firstly, there are differences in amplitude attenuation and phase shift between different transmitter-receiver pairs since the signals go through different paths. To solve this problem, a measurement of the switch matrix is also conducted. The measurement that needs to be performed is the $S_{21,i}^{3}$ of each path, including the switch boards and cables. Through this measurement, we can construct a series of complex coefficients that can be denoted as $C_{i}=S_{21,i}^{3}/S_{21,ref}^{3}$, *i* = 1, 2, ..., 24. Here, $S_{21,ref}^{3}$ corresponds to the reference path, in which the receiving antenna can be chosen as the one directly facing the transmitting antenna. Then the measured scattered data can be divided by *C_i_* for amplitude and phase compensation, i.e., $E_{mea,p}^{sca}=({\bar{E}}_{p}^{t}-{\bar{E}}^{i})/C$, *p* = 1, 2, …, 6, where $E_{mea,p}^{sca}$ denotes the measured data due to the *p-*th incidence after compensation, and C is a column vector whose elements are *C_i_*.

Secondly, there is a difference between the fields that we measured and the field that we used in the numerical calculation. It is easy to calibrate this due to the fact that $E_{ref}^{i}/E_{mea}^{i}=E_{cal}^{sca}/E_{mea}^{sca}$, where $E_{ref}^{i}$ denotes the simulated incident field at the receiving antenna directly facing the transmitting antenna, which can be considered as a reference. $E_{mea}^{i}$ and $E_{mea}^{sca}$ denote the measured incident fields by the receiving antenna directly facing the transmitting antenna and the scattered fields, respectively, and $E_{cal}^{sca}$ denotes the calibrated scattered field for the inversion algorithm.

Alternatively, the calibration of the measurement system can also be performed by measuring the scattered fields of a reference target. According to [51], this method may lead to more accurate results. However, much more effort is needed to carry out such a calibration. In this work, we chose the former one because the results we obtained after the simple calibration are acceptable.

### 4.3. Results

In our experiment, the scatterer is a 100-cm-long cylinder made of organic glass, whose nominal relative permittivity is around 3. The diameter of the cylinder is 5 cm.

In the first measurement, the cylinder is placed at the center of the receiving antennas. In the second measurement, the same cylinder is placed 5 cm off the center of the receiving antennas. The exact position in a Cartesian coordinate is (5cos30°, −5sin30°), or (4.33, −2.50) cm. In both measurements, the oblique incident angles are set as 15° and 30°, respectively, and the scattered fields are measured for six evenly-separated incident directions by rotating the turntable supporting the 2D object.

**Figure 11:**
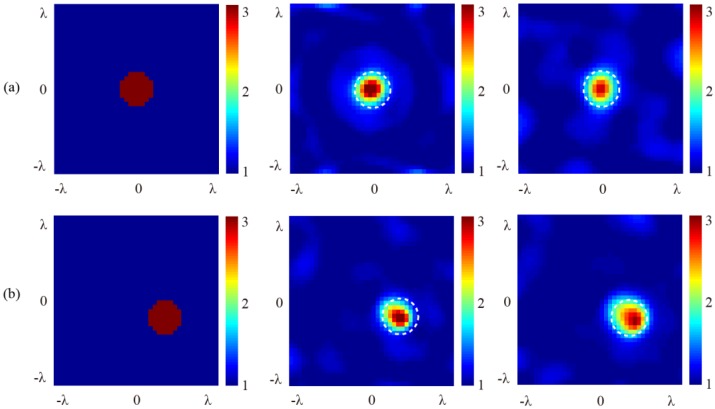
Retrieved results with experimental data for 15° and 30° angles of incidence. (**a**) Center-located target; and (**b**) off-center located target.

Figure 11 shows the original and the retrieved profiles for the target under test with different locations and angles of incidence. It is seen that in both cases, the scatters are successfully retrieved. Compared with the original profile, the retrieved results in Figure 11a for the center-located target are satisfactory for both angles of incidence. The location of the target, the relative permittivity in the central area and the overall cross-section profile satisfy the expectation. For the off-center located target, while the retrieved location and the relative permittivity of the target are satisfactory, the cross-section shapes are slightly distorted from the original profile. 

**Figure 12:**
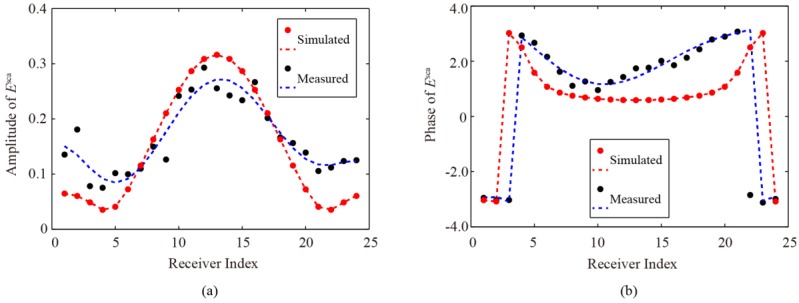
Comparison between simulated and measured scattered fields for a dielectric cylinder placed at the center of receiving antennas with 15° angle of incidence. (**a**) Amplitude; and (**b**) phase.

In order to investigate the source of errors that cause the differences between the original profiles and the retrieved results, we compare the magnitude and phase between the simulated and the measured data of the scattered fields. For demonstration, one group data for the off-centered cylinder are shown in Figure 12, where the dots represent the simulated and the measured data, and the dashed lines represent the corresponding fitting curves. It is seen that, the same as all experimental inversion problems, the measured data in our measurement also include errors and noise introduced by the environment and imperfections of the measurement system. Since the measured data, especially the measured amplitude data, notably deviate from their fitting curves, a degradation of the imaging quality would be inevitable.

In our experiment, the home-made setup was not placed in an anechoic chamber and, therefore, the reflections due to the uncovered part of the ground, the edges and gaps between the absorber screens, the metal surface of the turntable, and the supporting structures of the antennas would easily result in such errors. Even so, compared with existing experimental results for similar microwave imaging, the results shown in Figure 11 are, overall, satisfactory [35,39,40]. The notable errors shown in Figure 12 imply that if the measurement setup can be further improved and calibrated, much better imaging quality can be obtained.

### 4.4. Discussion

As stated, the motivation inspired this work is to improve the engineering practicability of SOM-based imaging for scatters that can be approximated as 2D objects, other than to improve the SOM itself. Therefore, we only used the very basic SOM in the inversion. If performance-enhanced SOMs can be used, better inversion results can be expected. Our analytical, simulation, and experimental results showed that by introducing TM oblique incidence and only detecting TM scattering, the simplified setup is able to bring advantages, like less complexity, simpler calibration, and faster computation, to the inverse imaging without notably losing the imaging quality. This agrees with [52], where the depolarizing effects are taken into account in solving qualitative inverse scattering problems for targets showing electric and magnetic contrast.

It is worth noting that in the proposed method, the incidence is always TM polarized. Therefore, our results can be different from those obtained with differently-polarized incidences [22,53]. Since the wave vector of the plane wave incidence has a component along the axis direction, couplings exist between the TE and TM modes [46]. In this case, the method proposed in [54], i.e., decomposing the plane wave into separate TE and TM modes, cannot be used. In fact, TE incidence can also be used in our case. However, as indicated in [22], the obtained results between TE and TM inversions are similar when scattered fields are sampled in the far field. Unlike the TM data, TE data should be decomposed into *x-y* components for the inversion. Therefore, from a data processing point of view, TE incidence does not have obvious advantages for our setup.

It is also worth noting that in the proposed method dedicated for 2D objects, 3D-2D modelling errors always exists. In this case, measures must be taken to ensure the error due to the 3D-2D modelling is controllable. For the proposed setup with oblique incidence, the longitudinal separation between the receiver and transmitter planes should be sufficiently smaller than the longitudinal section of the scatter. For a specific 2D scatter, this condition can be satisfied by adjusting the oblique angle, the detecting distance, and the operating frequency. For a smaller oblique angle, a shorter detecting distance and a higher frequency, the requirement of the length of the 2D object can be reduced.

Finally, the main purpose of this work is to investigate the possibility of performing microwave imaging on 2D objects under oblique incidences, rather than to obtain the best results. However, since the inverse scattering problem is essentially ill-posed, it would always require a highly accurate measurement system for microwave imaging. In this sense, further improvement of the proposed measurement setup deserves continued research efforts.

## 5. Conclusions

In conclusion, an inversion algorithm based on the SOM to solve inverse scattering problems under oblique microwave illumination is mathematically derived. A corresponding experimental setup combining the advantages of two types of conventional setups is designed and fabricated to verify the effectiveness of the proposed approach. Our results imply that for 2D objects, oblique microwave illumination will not cause a notable loss of information and, therefore, the quality of the microwave imaging will not be degraded compared with the conventional case under normal illumination, and can potentially be used in microwave imaging applications for 2D objects.

## Figures and Tables

**Table 1:** Error analysis for different 2D targets.

	Cylinder Profile		“Austria” Profile	
Incident Angle	TM Component	TE Component	TM Component	TE Component
15°	0.16%	2.95%	0.22%	9.31%
30°	0.56%	2.14%	0.28%	9.03%
45°	0.24%	1.49%	0.81%	9.34%
